# Characterization of Molecular Subtypes in Head and Neck Squamous Cell Carcinoma With Distinct Prognosis and Treatment Responsiveness

**DOI:** 10.3389/fcell.2021.711348

**Published:** 2021-09-14

**Authors:** Pei Zhang, Shue Li, Tingting Zhang, Fengzhen Cui, Ji-Hua Shi, Faming Zhao, Xia Sheng

**Affiliations:** ^1^Department of Stomatology, The First Affiliated Hospital of Zhengzhou University, Zhengzhou, China; ^2^Department of Stomatology, Union Hospital, Tongji Medical College, Huazhong University of Science and Technology, Wuhan, China; ^3^Hubei Province Key Laboratory of Oral and Maxillofacial Development and Regeneration, Wuhan, China; ^4^Key Laboratory of Environment and Health, Ministry of Education & Ministry of Environmental Protection, State Key Laboratory of Environmental Health (Incubation), School of Public Health, Tongji Medical College, Huazhong University of Science and Technology, Wuhan, China; ^5^Department of Hepatobiliary and Pancreatic Surgery, Henan Key Laboratory of Digestive Organ Transplantation, The First Affiliated Hospital of Zhengzhou University, Zhengzhou, China

**Keywords:** head and neck squamous cell carcinoma, molecular subgroups, gene expression, tumor immune microenvironment, prognosis

## Abstract

Head and neck squamous cell carcinoma (HNSCC) is one of the most aggressive malignancies with complex phenotypic, etiological, biological, and clinical heterogeneities. Previous studies have proposed different clinically relevant subtypes of HNSCC, but little is known about its corresponding prognosis or suitable treatment strategy. Here, we identified 101 core genes from three prognostic pathways, including mTORC1 signaling, unfold protein response, and UV response UP, in 124 pairs of tumor and matched normal tissues of HNSCC. Moreover, we identified three robust subtypes associated with distinct molecular characteristics and clinical outcomes using consensus clustering based on the gene expression profiles of 944 HNSCC patients from four independent datasets. We then integrated the genomic information of The Cancer Genome Atlas (TCGA) HNSCC cohort to comprehensively evaluate the molecular features of different subtypes and screen for potentially effective therapeutic agents. Cluster 1 had more arrested oncogenic signaling, the highest immune cell infiltration, the highest immunotherapy and chemotherapeutic responsiveness, and the best prognosis. By contrast, Cluster 3 showed more activated oncogenic signaling, the lowest immune cell infiltration, the lowest immunotherapy and chemotherapy responsiveness, and the worst prognosis. Our findings corroborate the molecular diversity of HNSCC tumors and provide a novel classification strategy that may guide for prognosis and treatment allocation.

## Introduction

Head and neck squamous cell carcinoma (HNSCC) is a primary malignant tumor that develops from the mucosal epithelium in the pharynx, larynx, and oral cavity, causing 600,000 new cases worldwide each year (Cancer Genome Atlas Network, 2015; Alsahafi et al., 2019; Canning et al., 2019). HNSCC is generally categorized into four subgroups, namely, basal, mesenchymal, atypical, and classical subtypes (Chung et al., 2004; Walter et al., 2013; Cancer Genome Atlas Network, 2015). Recent large-scale transcriptomic profiling has uncovered the molecular landscape of HNSCC, where genes involved in receptor tyrosine kinase (RTK)/RAS/PI3K signaling, cell cycle, cell death, and immunity signaling pathways are found frequently altered (Cancer Genome Atlas Network, 2015). These studies, on the one hand, underscored the complexity and heterogeneity of HNSCC tumors and, on the other hand, alerted that the prognosis and treatment strategy may be highly variable among tumors of different molecular features (Cancer Genome Atlas Network, 2015).

The current treatment of HNSCC includes surgery, radiation therapy, targeted therapy [epidermal growth factor receptor (EGFR)-targeting monoclonal antibody cetuximab], and chemotherapy with cytotoxic agents (such as cisplatin, methotrexate, gemcitabine, and bleomycin) (Johnson et al., 2020). Unfortunately, the current available treatments are largely ineffective and can cause severe toxicity (Alsahafi et al., 2019). Furthermore, approximately 50% of the patients experience recurrence (Leemans et al., 2018). Recent studies have shown that awakening the immune system with anti-PD1 and anti-CTLA4 therapies may be promising for recurrent/metastatic patients (Jie et al., 2015; Seiwert et al., 2016). However, a major limitation of these immune checkpoint inhibitors (ICIs) is the low responsive rate (Ferris et al., 2016; Burtness et al., 2019). Therefore, there is an urgent need to develop validated biomarkers to stratify HNSCC patients with potentially different survival outcome and treatment responsiveness and to reduce undesirable side effects.

To this end, we interrogated the publicly available gene expression datasets of HNSCC patients and screened for prognosis-related pathways by gene set variation analysis (GSVA) of paired tumor and normal samples. This led to the identification of three prognostic pathways and 101 core genes within which categorized the HNSCC patients into different subgroups with distinct prognosis. We then integrated the genomic information of 502 HNSCC samples to comprehensively evaluate the molecular features of different subtypes. Finally, we identified several potential immunotherapeutic and chemotherapeutic agents that may be of selected responsiveness for specific subtypes of HNSCC. Together, our findings corroborate the molecular diversity of HNSCC tumors while proposing a novel system of patient stratification with diverse prognosis and treatment option.

## Materials and Methods

### Dataset Selection and Preparation

The RNA-sequencing data (raw read count and FPKM normalized) and full clinical annotation of The Cancer Genome Atlas (TCGA)-HNSCC project (*n* = 546) were downloaded from Xena Public Data Hubs (Goldman et al., 2020). Somatic mutation data were obtained from the cBioPortal database.^1^ Gene expression profiles of GSE107591 (*n* = 46), GSE127165 (*n* = 114), GSE41613 (*n* = 97), GSE65858 (*n* = 270), and GSE427433 (*n* = 75) were downloaded from the Gene Expression Omnibus (GEO).^2^ The normal and tumor samples were already defined in the datasets analyzed, for example, for TCGA-HNSCC project (tumor = 402 and matched normal = 44), defining a sample as tumoral or healthy sample according to TCGA barcode, with tumors ranging from 01 to 09 and normal types from 10 to 19.

### Gene Set Variation Analysis and Functional Annotation

Pathway analyses were predominantly performed on the 50 hallmark gene sets (Liberzon et al., 2015). The enrichment scores of molecular pathways were evaluated by GSVA (Hanzelmann et al., 2013), which is commonly employed for estimating the variation in pathway and biological process activities in the samples of an expression dataset. To reduce pathway redundancies and overlaps, each gene set in 50 hallmark pathways was trimmed to only contain unique genes, and all genes associated with two or more pathways were excluded (Lambrechts et al., 2018). Most gene sets retained more than 70% of their associated genes. The GSVA was conducted on the gene profile through “GSVA” R packages, and differential pathways were identified using the R package “Limma” (Liu et al., 2015). Adjusted *p*-value < 0.05 was considered statistically significant. Univariate Cox analysis of overall survival (OS) was then performed to screen prognostic-related pathways.

Functional annotations were implemented by the “clusterProfiler” R package (Yu et al., 2012). Firstly, the log fold change of whole gene expression was acquired by paired differential expression analysis using DEseq2 R package (Love et al., 2014) between paired tumor and normal samples. Next, the gene set enrichment analysis (GSEA) was applied to verify the activation of the mTORC1 signaling, unfolded protein response (UPR) pathway, and UV response UP, which led to the identification of 101 common core genes from TCGA, GSE107591, and GSE127165. Gene Ontology (GO) and Kyoto Encyclopedia of Genes and Genomes (KEGG) analyses were further employed for functional annotations of these core genes. Adjusted *p*-value less than 0.05 was considered as statistically significant.

### Identification of Core Gene Expression-Based Subtypes

To functionally elucidate the biological characteristics of the core genes from mTORC1 signaling, UPR pathway, and UV response UP, unsupervised clustering was performed using the “ConsensusClusterPlus” R package (Wilkerson and Hayes, 2010) to classify HNSCC patients into different subtypes. We first normalized the expression of each gene by log transform across all tumors before applying a robustified *z*-score transformation (median-centered and mad-scaled) per sample. Then we extracted the normalized expression data of the 101 core genes for consensus cluster analysis. And 80% item resampling, 100 resamplings, a maximum evaluated *K* of 8, and ward2 algorithm (Murtagh and Legendre, 2014) were selected for clustering. The cumulative distribution function (CDF) and consensus heatmap were used to assess the optimal *K*.

### Estimation of Tumor Purity and Immune Cell Type Fractions

R package “estimate” was used to evaluate the stromal score, immune score, and tumor purity of each patient (Yoshihara et al., 2013). The immune infiltration of 22 immune cell types was explored using the CIBERSORT algorithm, in combination with the LM22 signature matrix (Newman et al., 2015).

### Therapeutic Response Prediction

To identify potential targets for immunotherapy, the gene expression profile of T-cell signaling pathway was examined (Chen et al., 2017). Furthermore, the Tumor Immune Dysfunction and Exclusion (TIDE) algorithm (Jiang et al., 2018) and immune-related genetic prognostic index (IRGPI) score (Chen et al., 2021) were used to predict ICI response for HNSCC. Besides, unsupervised subclass mapping method (SubMap) algorithms (Hoshida et al., 2007) were used to predict PD-1 and CTLA4 in three subtypes identified by us with another published dataset containing 47 melanoma patients who responded to immunotherapies (Roh et al., 2017).

“pRRophetic” R package was used to predict half-maximal inhibitory concentration (IC50) for each sample by ridge regression, and the prediction accuracy was evaluated by 10-fold cross-validation based on the Genomics of Drug Sensitivity in Cancer (GDSC) training set (Geeleher et al., 2014). The GDSC^3^ is one of the largest publicly available pharmacogenomics databases. All parameters were set by the default values with transcripts per kilobase million data. *p*-Value < 0.05 was considered statistically significant.

### Statistical Analysis

All data analyses were performed in the R platform (x64, version 4.0.2). Student’s *t*-test or Wilcoxon rank-sum test was performed to compare continuous variables between two groups. Chi-square test or Fisher’s exact test was used for categorical data. One-way ANOVA or Kruskal–Wallis tests were used to conduct difference comparisons of three groups. Kaplan–Meier analysis with log-rank tests was performed to assess survival difference between groups *via* “survminer” R package. The mutation landscape in HNSCC patients with different subtypes was exhibited using “maftools” R package. All statistical *p*-values were two-sided, and *p* < 0.05 was considered statistically significant.

## Results

### Identification of the Prognostic Pathways and Core Genes for Overall Survival in Head and Neck Squamous Cell Carcinoma

**FIGURE 1:**
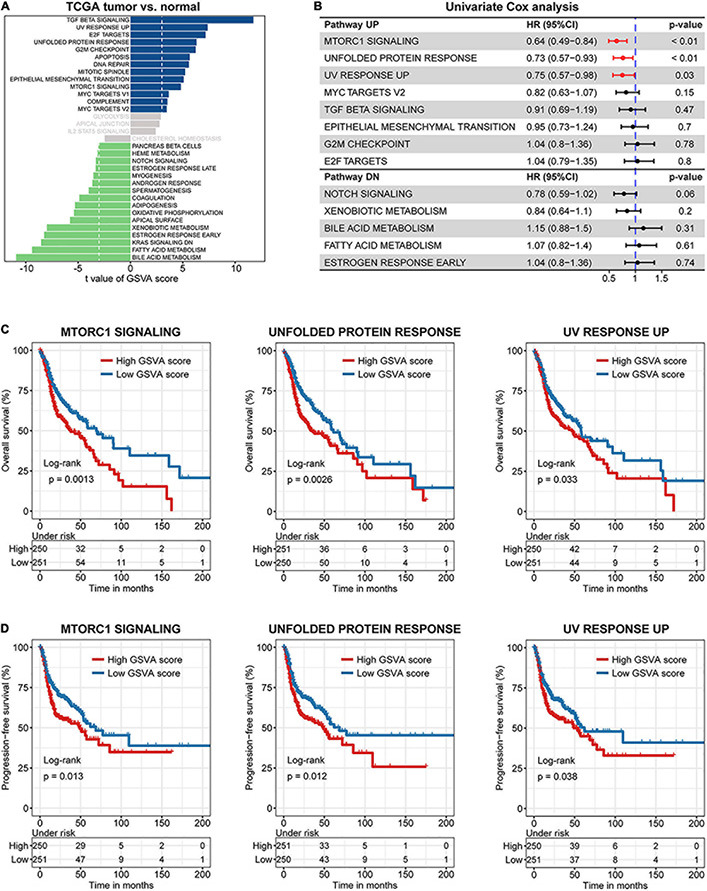
The major prognostic pathways in head and neck squamous cell carcinoma (HNSCC) identified by gene set variation analysis (GSVA). **(A)** Differences in pathway activities scored per sample by GSVA between paired tumor and normal HNSCC in The Cancer Genome Atlas (TCGA) (*n* = 44). Shown are *t*-values from a linear model, corrected for patient of origin. dn, down; UV, ultraviolet; v1, version 1; v2, version 2. **(B)** Univariate Cox regression analysis among various hallmarks of cancer. Kaplan–Meier curves of overall **(C)** and progression-free **(D)** survival for HNSCC patients in the two groups divided by GSVA pathway score.

To screen for robust oncogenic signaling across different HNSCC cohorts, we applied GSVA based on the expression profile and identified several differentially activated pathways between paired tumor and normal samples in TCGA (*n* = 44), GSE107591 (*n* = 23), and GSE127165 (*n* = 57) datasets (Figure 1A and Supplementary Figure 1A). A Venn diagram showed the 13 commonly altered oncogenic pathways, including eight upregulated pathways (such as mTORC1 signaling) and five downregulated pathways (such as notch signaling) (Supplementary Figure 1B). Univariate Cox regression of OS based on GSVA scores in TCGA HNSCC cohort (patients *n* = 502) then identified three candidate prognostic pathways with statistical significance, including mTORC1 signaling (HR = 0.65, 95% CI = 0.49–0.84, *p* < 0.01), UPR pathway (HR = 0.73, 95% CI = 0.57–0.93, *p* < 0.01), and UV response UP (HR = 0.75, 95% CI = 0.57–0.98, *p* = 0.03) (Figure 1B). Interestingly, Kaplan–Meier survival analysis showed that the OS and progression-free survival (PFS) of the high GSVA score group was significantly shorter than those of the low GSVA score group in TCGA cohort (Figures 1C,D).

**FIGURE 2:**
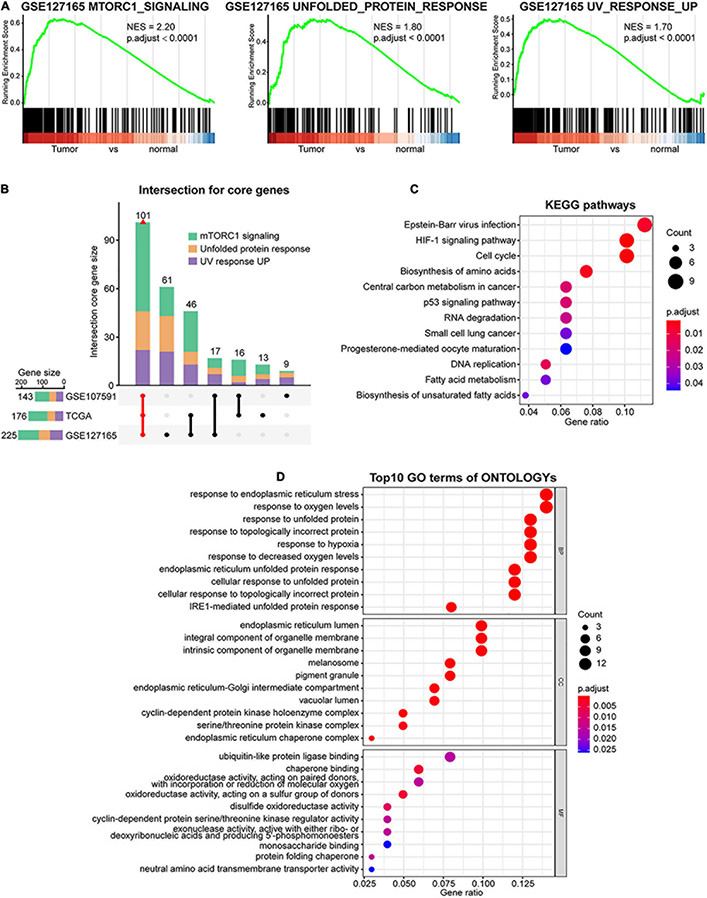
Identification of the core genes in mTORC1 signaling, unfolded protein response (UPR), and UV response UP gene sets. **(A)** Gene set enrichment analysis (GSEA) shows that mTORC1 signaling, UPR, and UV response UP are significantly enriched in GSE127165 cohort. **(B)** Intersection of core genes based on GSEA from three databases is indicated in red. Kyoto Encyclopedia of Genes and Genomes (KEGG) **(C)** and Gene Ontology (GO) **(D)** analysis of the common core genes. BP, biological processes; CC, cellular components; MF, molecular function.

To further verify the activation of the three oncogenic pathways in HNSCC samples, GSEA was performed between paired tumor and normal samples in TCGA, GSE107591, and GSE127165 cohorts. As expected, the three oncogenic pathways were all significantly enriched in tumor samples (Figure 2A and Supplementary Figure 1C). Furthermore, 101 significantly deregulated genes within the three pathways were identified, 11 of which belonged to multiple pathways due to their functional diversity (Figure 2B and Supplementary Table 1). Interestingly, KEGG analyses of the core genes showed the highest score of Epstein–Barr virus (EBV) infection (Figure 2C), one of the established risk factors leading to nasopharyngeal carcinoma (Fernandes et al., 2018). This indicates that the core genes selected are highly relevant to HNSCC. Besides, KEGG and GO analyses also demonstrated that the core genes were involved in processes such as endoplasmic reticulum (ER) stress, hypoxia, cell cycle, DNA damage, and repair (Figures 2C,D).

### Identification of Three Distinct Subtypes of Head and Neck Squamous Cell Carcinoma

**FIGURE 3:**
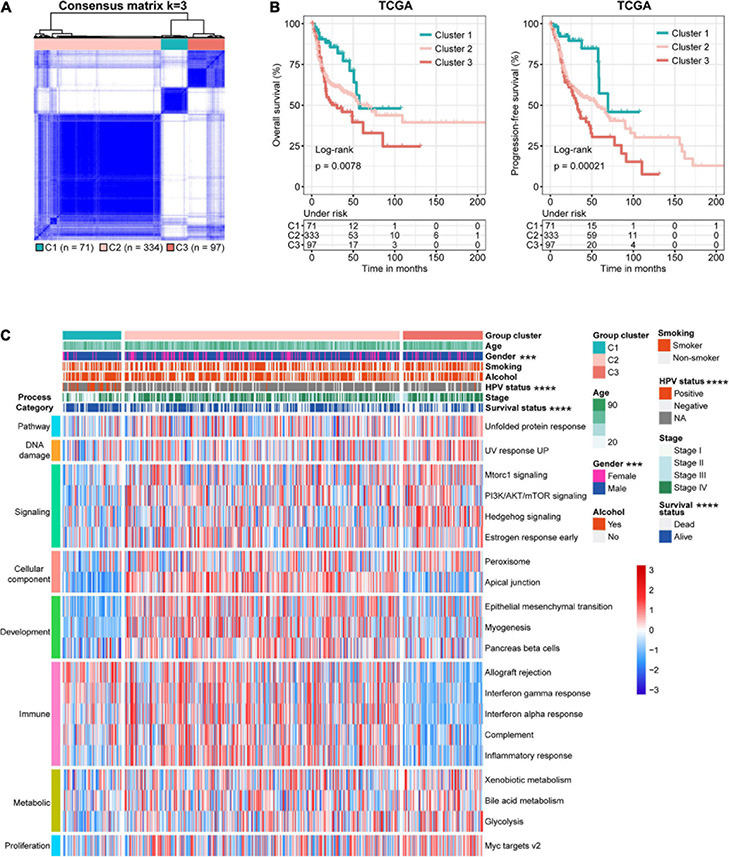
Identification of three molecular subtypes of head and neck squamous cell carcinoma (HNSCC) in The Cancer Genome Atlas (TCGA) discovery dataset. **(A)** Consensus matrix heatmaps (*k* = 3) of common core genes in The Cancer Genome Atlas (TCGA) cohort. **(B)** Kaplan–Meier curves of overall (left, OS) and progression-free (right, PFS) survival for HNSCC patients in different subtypes. **(C)** Heatmap of differentially activated pathways based on gene set variation analysis (GSVA) score and clinicopathological features of the three subtypes.

In addition to identifying key genes and pathways, cancer subtyping is critical to improving personalized treatment (Berger et al., 2018). Toward this goal, we utilized unsupervised consensus clustering based on the expression profile of these core genes, where a total of 502 patients from TCGA cohort were clustered into three subtypes, namely, Cluster 1 (C1, *n* = 71), Cluster 2 (C2, *n* = 334), and Cluster 3 (C3, *n* = 97) (Figure 3A and Supplementary Figure 2A). Upon comparison of the survival rates among the three subtypes, we found that the C1 patients showed the best OS and PFS (Figure 3B). To validate this correlation, we performed the same analysis in additional HNSCC datasets, where a distinct survival rate of OS or PFS showed in different subtypes in GSE41613 (*n* = 97), GSE65858 (*n* = 270), and GSE42743 (*n* = 74) (Supplementary Figures 2B–D). Interestingly, the C1 subtype displayed the lowest GSVA score of several pathways that are known to be oncogenic or activated in cancers, such as mTORC1 signaling, UPR, and UV response UP pathways (Supplementary Figures 2E, 3A). Furthermore, according to the differential analysis of pathway GSVA scores, the C1 and C2 subgroups showed stronger enrichment of immune pathways, such as inflammatory response, and lower enrichment of oncogenic processes, such as DNA damage, and metabolic and proliferation pathways, than did C3 subgroup (Figure 3C). We also noted no difference in age, smoking status, alcohol history, and cancer stage among different subtypes in TCGA cohort, despite more male and human papillomavirus (HPV)-positive patients in the C1 subtype (Figure 3C and Supplementary Figure 3B). Taken together, these results demonstrate that the status of 101 core genes identified from the UPR, UV response UP, and mTORC1 signaling is able to distinguish the HNSCC patients with different molecular features and survival outcomes.

### Comparison of the Mutational Profile and Burden Between the Three Subtypes

**FIGURE 4:**
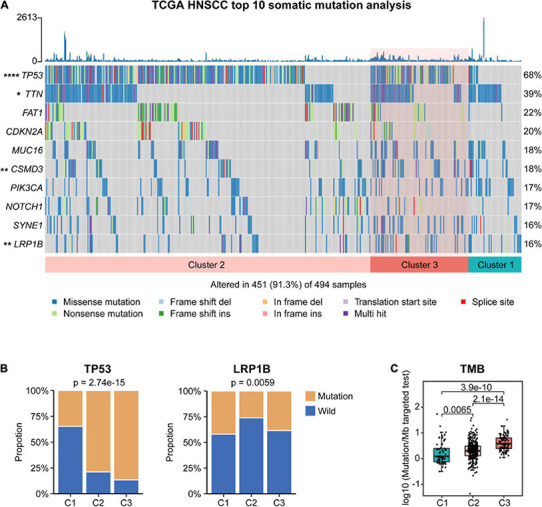
Association between different molecular subtypes and tumor somatic mutation in The Cancer Genome Atlas (TCGA) cohort. **(A)** Top 10 highly mutated genes in head and neck squamous cell carcinoma (HNSCC) subtypes. **(B)** The proportion of *TP53* and *LRP1B* mutation in different subtypes. **(C)** Tumor mutational burden (TMB) of different subtypes.

To investigate the difference in somatic mutation among these subtypes, we examined the mutect2-processed mutation dataset in TCGA. As shown in Figure 4A, the landscape of top 10 genes with the most frequent genomic alterations in HNSCC is displayed. Four of the top 10 mutation genes (*TP53*, *TTN*, *CSMD3*, and *LRP1B*) showed a distinct mutation rate among the three subtypes. For instance, the canonical tumor suppressor gene *TP53* was more frequently mutated in C2 and C3 patients than in C1 patients (*p* = 2.74e−15, Chi-square test), whereas another frequently deleted gene in HNSCC *LRP1B* showed a lower mutation rate in C2 compared with C1 and C3 (*p* = 0.0059, Chi-square test) (Figure 4B). Compared with C1 and C2, C3 showed significantly higher tumor mutational burden (TMB) (Figure 4C), a parameter correlated with unfavorable immune expression signatures and poor clinical outcomes in HNSCC patients (Chen et al., 2019; Eder et al., 2019).

### Characterization of Immunological Features Among Different Subtypes

**FIGURE 5:**
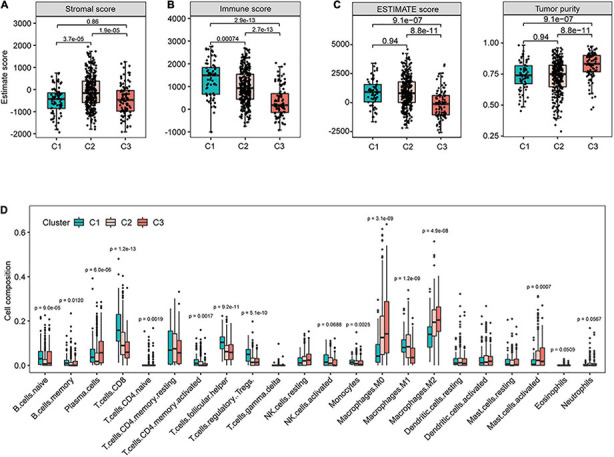
Identification of immune characteristics for different subtypes in The Cancer Genome Atlas (TCGA) cohort. The distribution of stromal score **(A)**, immune score **(B)**, ESTIMATE score, and tumor purity **(C)** for patients in different subtypes. **(D)** The infiltrating levels of 22 immune cell types in different subtypes in The Cancer Genome Atlas (TCGA) cohort.

Given that these subtypes exhibited marked difference in immune-related pathways and TMB, we further explored the characteristics of the tumor microenvironment within different subtypes. To do so, we first examined the distribution of stromal score, immune score, and tumor purity of different subgroups by computing the ESTIMATE algorithm (Yoshihara et al., 2013). C2 showed the highest stromal score (Figure 5A), suggesting the highest stromal content in C2 tumors. C2 subtype also exhibited higher immune scores than C3 (Figure 5B), indicative of the higher infiltration of immune cells. Moreover, the ESTIMATE score (combining the stromal and immune scores) of C3 was significantly lower compared with that of other subtypes, whereas its tumor purity was the highest (Figure 5C). These data suggest that low immune infiltration may be a phenotypic feature of C3 tumors.

To gain further insight in this regard, we performed CIBERSORT method to estimate the differences in the infiltration of 22 common immune cell types. In line with the immune score results, C3 tumors showed the lowest proportions of lymphocyte infiltration including CD8^+^ T cells, CD4 memory-activated T cells, regulatory T cells, and activated NK cells (Figure 5D). Tumor-associated macrophages polarize into different subtypes that may either promote (M2 subtype) or inhibit (M1 subtype) tumor growth (Anderson et al., 2021). We found that C3 had the highest proportions of resting M0 and polarized M2 macrophages and the lowest level of M1 macrophages (Figure 5D). Together, these findings suggest potentially diverse immunological profiles among different subtypes of HNSCC tumors.

### Prediction of Potentially Responsive Treatment Strategies for Different Subtypes

**FIGURE 6:**
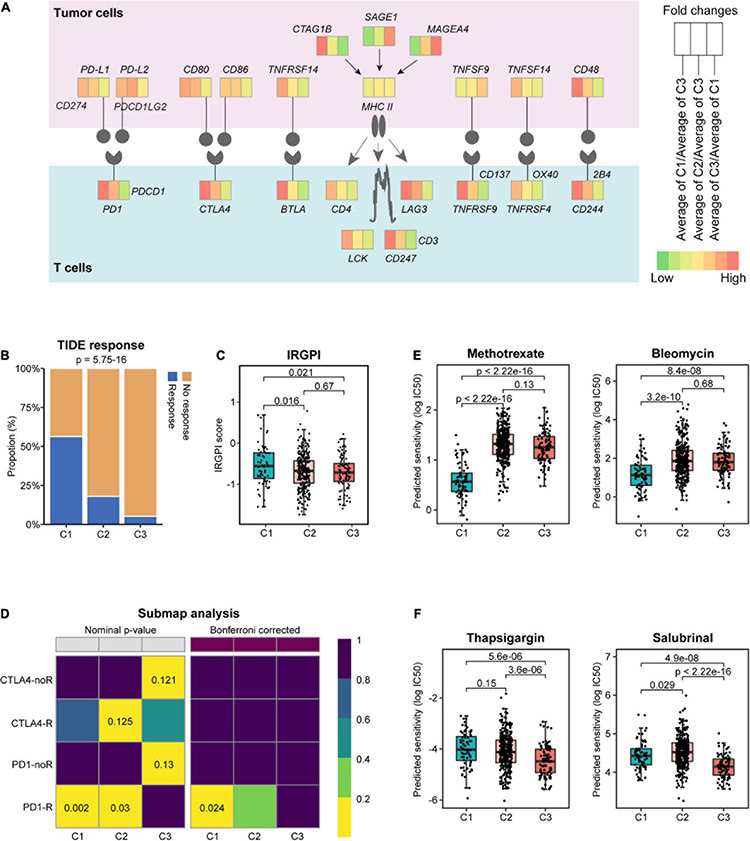
Prediction of response to immunotherapeutic and therapeutic agents for different subtypes. **(A)** Diagram of the immune checkpoint pathway, comparing tumors in different subtypes. Tumor Immune Dysfunction and Exclusion (TIDE) response **(B)** and immune-related genetic prognostic index (IRGPI) score **(C)** in different subtypes. **(D)** Subclass mapping method (SubMap) analysis predicted the sensitivities of different subtypes to the four immune checkpoint inhibitors. noR, no response; R, response. The box plots depict the estimated IC50 for methotrexate, bleomycin **(E)**, and thapsigargin and salubrinal **(F)**.

Building upon these findings, we speculate that these subtypes of HNSCC tumors may respond to different therapies. To identify potential targets for immunotherapy, the gene expression profile of T-cell signaling pathway was examined (Chen et al., 2017). Interestingly, we found that the expression of several canonical immune checkpoints was markedly elevated in C1 than in C3 (Figure 6A and Supplementary Figure 4A), such as *CD274* (PD-L1), *PDCD1LG2* (PD-L2), *PDCD1* (PD1), and *CTLA4* (CD152), potentiating the effectiveness of ICI in C1 tumors.

To validate this, we assessed the potential clinical efficacy of immunotherapy in different subtypes by TIDE, whose score can be predictive of the potential for immune evasion (Jiang et al., 2018). Our analysis found that C1 tumors had the highest response rate to immunotherapy (Figure 6B) and the lowest TIDE score (Supplementary Figure 4B). In addition to the TIDE prediction, IRGPI score was recently proposed as a promising immune-related prognostic biomarker in HNSCC (Chen et al., 2021). In keeping with the previous findings, C1 tumors showed significantly higher IRGPI score than C2 and C3 tumors (Figure 6C). These results suggested that patients of C1 subtype were more likely to benefit from ICI therapy than those of C2 and C3 subtypes. To examine this in a more detailed manner, SubMap analysis was conducted, and the results indicated that C1 tumors were more likely to respond to anti-PD1 therapy (Figure 6D; Bonferroni-corrected *p* = 0.024).

Furthermore, we assessed the potential responsiveness of the subtypes to the existing HNSCC drugs by estimating the drug IC50 for each sample in TCGA cohort through 10-fold cross-validation based on the GDSC training set (Geeleher et al., 2014). Interestingly, compared with C2 and C3 tumors, C1 showed markedly lower estimated IC50 of chemotherapeutic drugs, such as methotrexate, bleomycin, cisplatin, gemcitabine, and entinostat (Figure 6E and Supplementary Figure 4C), suggesting a selective sensitivity of these tumors to chemotherapy. Besides these clinical drugs, further interrogation of the GDSC database found that C2 tumors may be responsive to the HSP90 inhibitor luminespib, and the MEK 1/2 inhibitor selumetinib (Supplementary Figure 4D), whereas C3 tumors were predicted to be sensitive to agents disrupting ER homeostasis, such as the SERCA pump inhibitor thapsigargin and the eIF2α phosphatase inhibitor salubrinal (Figure 6F), as well as the mTOR inhibitor AZD8055 (Supplementary Figure 4D).

### Integration of the Three Subgroups With Other Immune and Molecular Classes

**FIGURE 7:**
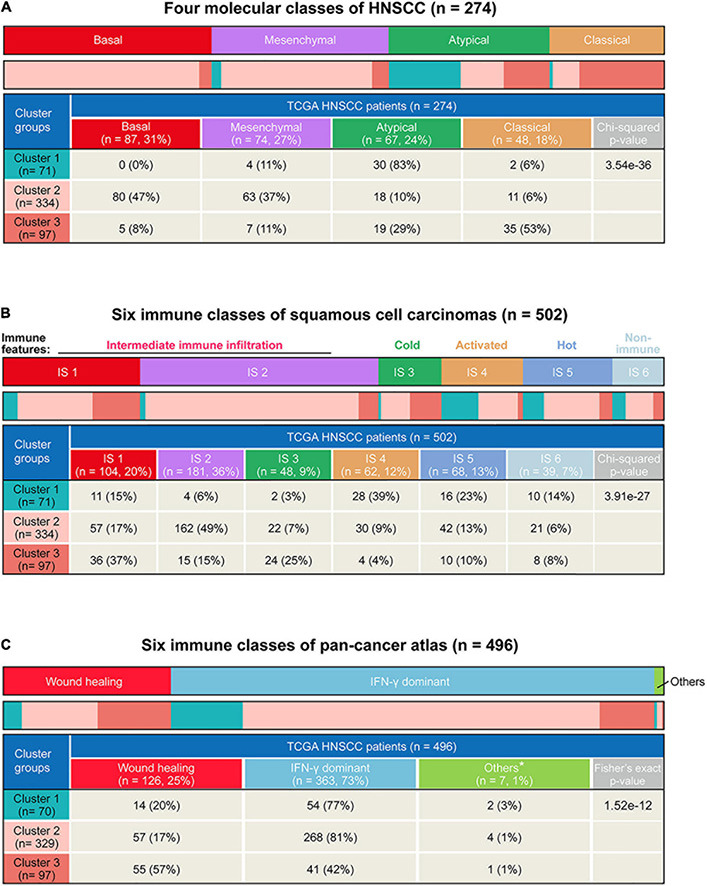
Comparison of the three subtypes in our study with existing immune and molecular classes. **(A)** Heatmap and table showing distribution of four head and neck squamous cell carcinoma (HNSCC) molecular classes (basal, mesenchymal, atypical, and classic) in different subtypes. **(B)** Heatmap and table showing the distribution of pan-squamous cell carcinomas (SCC) immune classes (IS1, IS2, IS3, IS4, IS5, and IS6) between the three subtypes. **(C)** Heatmap and table showing distribution of six pan-cancer immune classes in different subtypes. *Other immune classes include inflammatory, lymphocyte depleted, immunologically quiet, and TGF-β dominant.

Head and neck squamous cell carcinoma tumors can be grouped as basal, mesenchymal, atypical, and classical subtypes according to statistically significant chromosomal gains and losses and differential cell of origin expression patterns (Chung et al., 2004; Walter et al., 2013; Cancer Genome Atlas Network, 2015). The basal class demonstrates inactivation of *NOTCH1* with intact oxidative stress signaling and fewer alterations of chromosome 3q. The mesenchymal subtype, characterized by either the presence of fibroblasts or a strong desmoplastic response, displays common alterations in innate immunity genes. The atypical class is a less aggressive subtype associated with a strong immune signature and a lack of chromosome 7 amplifications. In addition, the classical subtype shows high *TP53* mutation rate and alterations of oxidative stress genes. Based on the subtyping of HNSCC tumors, we detected the highest proportion of the atypical class (83%) within C1 (Figure 7A). Moreover, C2 was a mixed subtype composed of mesenchymal and basal samples, whereas C3 had more classical samples (*p* = 3.54e−36, Chi-squared test) (Figure 7A).

A squamous cell carcinoma immune subtype classification has described the immune landscape of HNSCC according to consensus clustering of immune-related gene expression profiles and has summarized six immune subtypes (Li et al., 2019). In line with the previous immunological profile analysis, there were more IS4 (immune-activated phenotype, 39%) and IS5 (immune-hot phenotype, 23%) classes in C1 while more IS1 (immune-suppressive phenotype, 37%) and IS3 (immune-cold phenotype, 25%) classes in C3 (*p* = 3.91e−27, Chi-square test) (Figure 7B).

Lastly, we also integrated our results with the six pan-cancer immune subtypes (Thorsson et al., 2018). Among these, the Wound healing class is defined by elevated expression of angiogenic genes and a Th2 cell bias to the adaptive immune infiltrate, while the IFN-γ dominant class is defined by the highest M1/M2 macrophage polarization and a strong CD8 signal. We found that C1 (77%) and C2 (81%) had significantly higher proportion of IFN-γ dominant class, whereas C3 showed the highest proportion of the Wound healing class (*p* = 1.52e−12, Fisher’s exact test) (Figure 7C). Taken together, by comparing our subtypes with previously established molecular and immunological classifications, these results corroborated that the C1 tumors were characterized by an active immune response and lower tumor aggressiveness, while the C3 tumor was characterized by an immune-suppressive response and higher aggressiveness.

## Discussion

**FIGURE 8:**
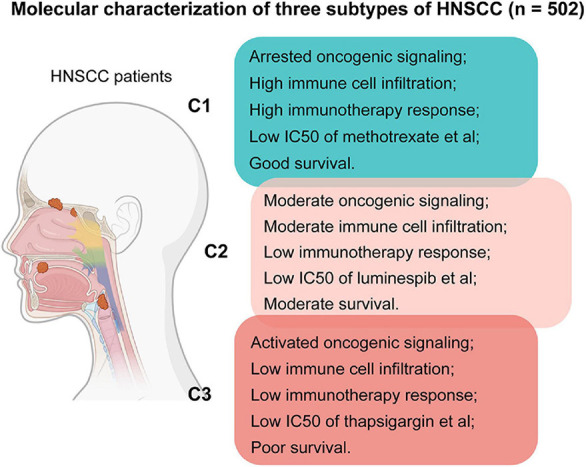
Summary of features for patients of the three subtypes in this study.

Head and neck squamous cell carcinoma is an aggressive malignancy, whose highly heterogeneous nature leads to disparities in prognosis and therapeutic response irrespective of clinical stage (Cancer Genome Atlas Network, 2015; Canning et al., 2019). Therefore, identifying biomarkers to stratify patients into clinically meaningful subtypes and selecting effective targeted therapies for different subtypes has become the primary focus of the field (Berger et al., 2018). In this study, by comprehensive bioinformatics analysis of the published datasets, we identified three subtypes of HNSCC tumors with distinct molecular features and survival outcomes, and we proposed potentially suitable therapeutic agents for specific subtypes (Figure 8), which may facilitate patient stratification and tailored treatment strategies in HNSCC.

Recently, several large-scale comprehensive studies have advanced our understanding in the molecular landscape of HNSCC and uncovered frequently altered genes and pathways (Chung et al., 2004; Walter et al., 2013; Cancer Genome Atlas Network, 2015). To identify the gene sets that are relevant to prognosis, we compared the paired tumor and normal samples from these publicly available datasets by GSVA and found that mTORC1 signaling, UPR, and UV response UP were the most significantly enriched upregulated pathways (*p* < 0.05). As a central regulator of metabolism, activation of mTORC1 signaling has been linked with poor prognosis in various malignancies, including HNSCC (Tan et al., 2019). Hyper-activated mTORC1 phosphorylates 4E-BP1, which stabilizes p21 and affects HNSCC clinical outcome (Llanos et al., 2016). Clinical trials of mTORC1 inhibitors used either alone or in combination with chemotherapy or radiotherapy showed encouraging results in HNSCC. Therefore, from a precision treatment standpoint, identifying the patients who may benefit from mTORC1 inhibitors is of clinical significance (Tan et al., 2019). Genes involved in the UPR and UV response are vital for maintaining proteostasis and genome stability (Mansilla et al., 2013; Strozyk and Kulms, 2013; Wang et al., 2019; Zhang et al., 2020). Previous evidence has functionally implicated these homeostatic mechanisms in HNSCC by regulating key tumor biology processes including cancer cell survival and therapy resistance, while their activities have also been associated with HNSCC prognosis (Pluquet and Galmiche, 2019; Psyrri et al., 2021). Furthermore, by overlapping the genes in these three pathways across different cohorts, we identified 101 core genes that were able to subgroup the patients with distinct survival outcomes and molecular profiles. Patients with the best prognosis (C1) were associated with arrested oncogenic pathways, such as PI3K/AKT/mTOR signaling and Myc targets. These results, at least in concept, provide a novel strategy to classify the HNSCC patients with divergent survival outcomes. Further refinement of the gene set is needed to generate biomarkers that may be of clinical utility.

To gain further biological insight into the molecular characteristics of three subtypes, we examined the mutect2-processed mutation dataset in TCGA, and we identified four commonly altered genes in HNSCC (*TP53*, *TTN*, *CSMD3*, and *LRP1B*) with distinct mutation rate among the three subtypes. As the single most commonly reported genetic abnormality in cancers, *TP53* aberration is also a molecular hallmark of HNSCC. The reported alteration frequency in HNSCC ranged from 20 to 90%, depending on the methodologies used, types of tumor materials sampled, and heterogeneity of tumor sites examined (Zhou et al., 2016). *TP53* encodes for a nuclear phosphoprotein that acts as a sequence-specific transcription factor and is involved in a plethora of processes, including cell cycle regulation, cellular response to DNA damage, senescence, and apoptosis (Zhou et al., 2016). *LRP1B* belongs to the gene family of low-density lipoprotein receptor and is also reputed as a tumor suppressor gene. Its frequent mutation has been observed in several cancer types, including HNSCC, and is associated with tumor HPV status and response to ICI (Cao et al., 2021). *TTN* encodes the large muscle protein titin that is primarily responsible for stabilization of cytoskeletal filaments (Lee et al., 2021), while *CSMD3* encodes for a large transmembrane protein with CUB and sushi multiple domains (Shimizu et al., 2003). Despite the frequent aberrations observed in HNSCC, neither of their functional role is clear so far. Studies even indicate that high alteration frequencies of *TTN* and *CSMD3* are likely due to heterogeneous mutation rates at different chromosome locations (Lawrence et al., 2013, 2014). Immunotherapies are a valuable addition to the arsenal of HNSCC treatments. However, the complexity in tumor microenvironment and immunity across different tumors has hindered its effectiveness in HNSCC. Here, we evaluated the immune-related parameters of different subtypes by computing multiple previously established algorithms. The results showed that C1 patients with the best prognosis displayed the highest immune, ESTIMATE, and IRGPI scores and the lowest TIDE response and score. The composition of activated cytotoxic T cells and NK cells was the highest in C1, whereas that of M2 macrophage was the lowest. These findings suggest that C1 subtype may represent the immunologically “hot” tumors that would likely benefit from ICI. In keeping with this, our drug response analysis predicted that C1 tumors were significantly more sensitive to chemotherapeutic and immunotherapeutic agents. By contrast, our analysis suggested that the immunologically “cold” C3 tumors may not directly benefit from either chemotherapy or immunotherapy but may be selectively sensitive to compounds disrupting ER homeostasis. This is consistent with the observation that UPR activity is the highest in these tumors. It would be of interest to dissect the role of different UPR branches in HNSCC experimentally and to evaluate the therapeutic value using branch-specific inhibitors that have been under extensive preclinical and clinical development (Hetz et al., 2019; Sheng et al., 2019; Torres et al., 2019).

## Conclusion

In conclusion, as an effort toward patient stratification and individualized treatment, we performed comprehensive bioinformatics analysis of the published datasets of HNSCC, and we proposed a novel classification strategy that can effectively categorize patients with different survival outcomes, molecular features, and immunological profiles. We also identified potentially suitable drugs and therapeutic strategies for each subtype. Our findings gain new insight into the heterogeneous nature of HNSCC and offer priorities for future experimental investigations.

## Data Availability Statement

The datasets presented in this study can be found in online repositories. The names of the repository/repositories and accession number(s) can be found in the article/Supplementary Material.

## Author Contributions

PZ, SL, and FZ designed the study and conducted the bioinformatics analysis. TZ and J-HS helped in the data analysis and revised the manuscript. XS and FZ wrote the manuscript and supervised the project. All authors read and approved the final manuscript.

## Conflict of Interest

The authors declare that the research was conducted in the absence of any commercial or financial relationships that could be construed as a potential conflict of interest.

## Publisher’s Note

All claims expressed in this article are solely those of the authors and do not necessarily represent those of their affiliated organizations, or those of the publisher, the editors and the reviewers. Any product that may be evaluated in this article, or claim that may be made by its manufacturer, is not guaranteed or endorsed by the publisher.

## Footnotes

1
http://www.cbioportal.org/datasets

2
https://www.ncbi.nlm.nih.gov/gds

3
https://www.cancerrxgene.org/
