# Supplementary material for: Characterization of Molecular Subtypes in Head and Neck Squamous Cell Carcinoma With Distinct Prognosis and Treatment Responsiveness
Source: Front Cell Dev Biol. 2021 Sep 14;9:711348. doi: 10.3389/fcell.2021.711348 (PMC8476885; doi:10.3389/fcell.2021.711348)
Supplement: Supplementary file 2 [file Data_Sheet_2.docx]

Supplementary Material

**
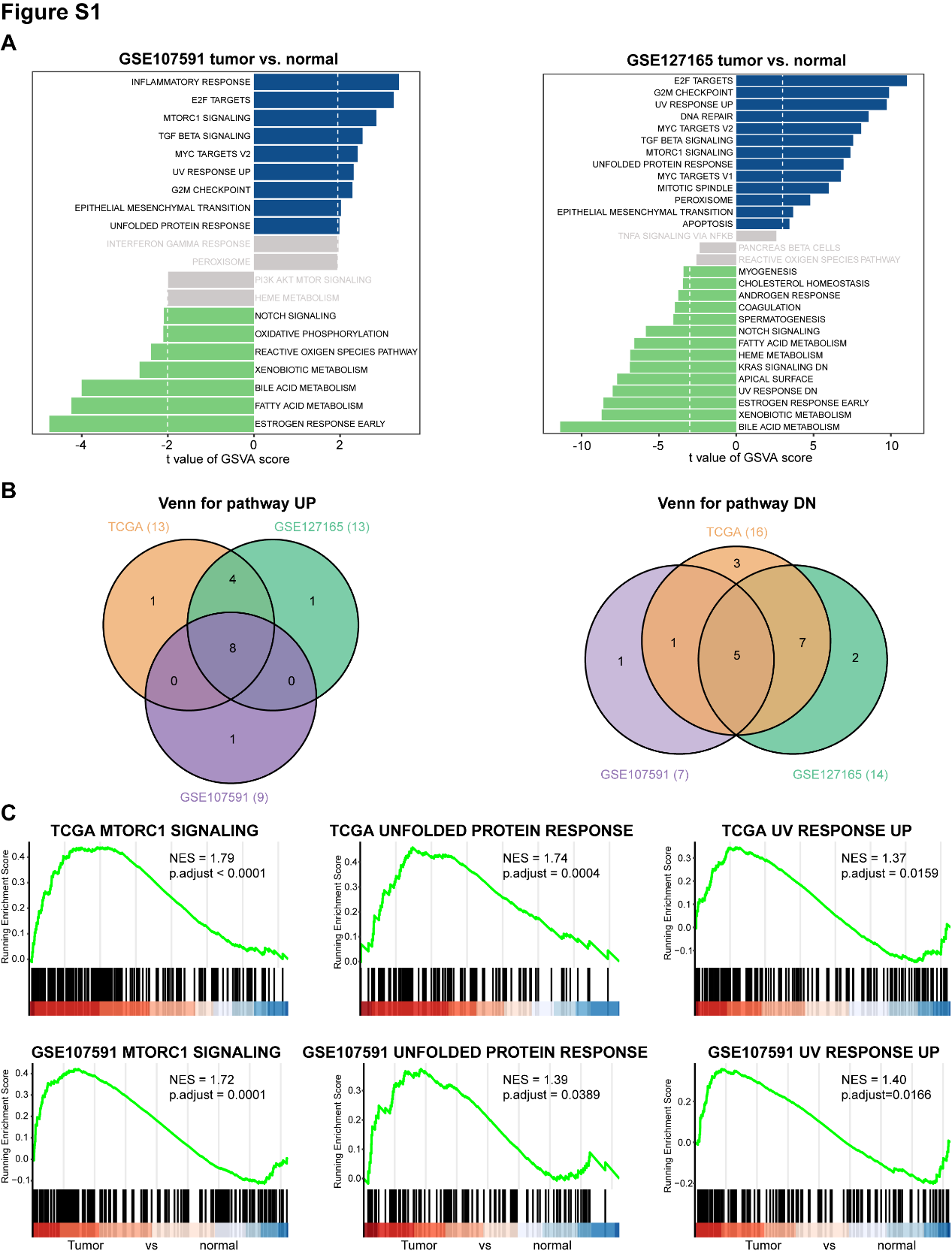
**

**Figure S1. The primary risk factors for HNSCC.**

A. Differences in pathway activities scored per sample by GSVA between paired tumor and normal HNSCC in GSE107591 (left, n = 23) and GSE127165 (right, n= 57) cohorts. Shown are t values from a linear model, corrected for patient of origin. dn, down; UV, ultraviolet; v1, version 1; v2, version 2. B. Veen diagram of upregulated (left) and downregulated (right) pathways based on the three datasets. C. GSEA shows that mTORC1 signaling, UPR and UV response UP are significantly enriched in TCGA and GSE107591 cohorts.

**
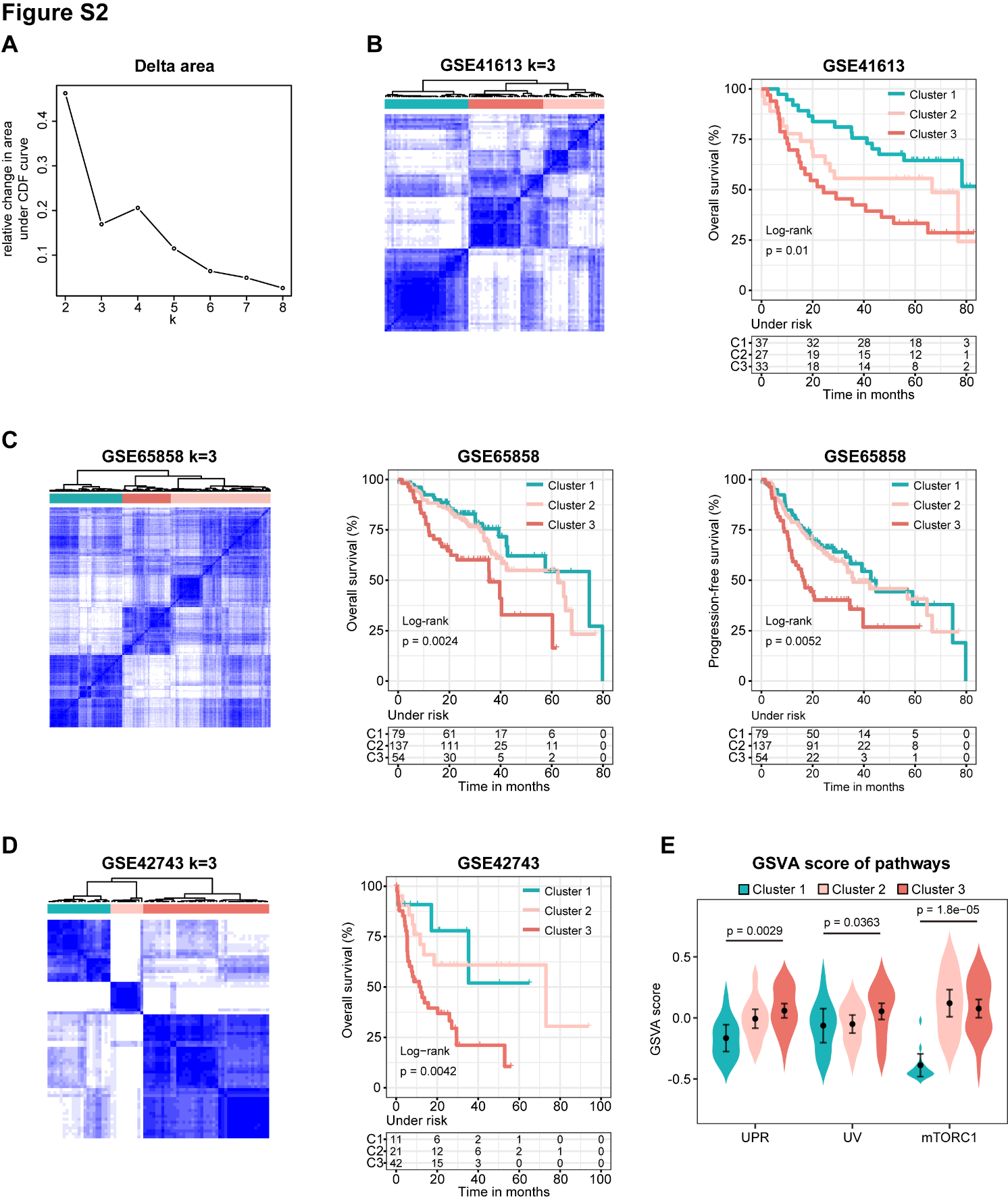
**

**Figure S2. The core genes classify three molecular subtypes of HNSCC in the GEO validation datasets.**

A. The relative changes in area under the cumulative distribution function (CDF) curve with increasing k. B-D. Consensus matrix heatmaps (k = 3) and prognostic analysis in the GSE41613 (B, n = 97), GSE65858 (C, n = 270), and GSE427433 (D, n = 75) cohorts. E. Distribution of GSVA score of UPR, UV response UP and mTORC1 signaling pathway across different subtypes.

**
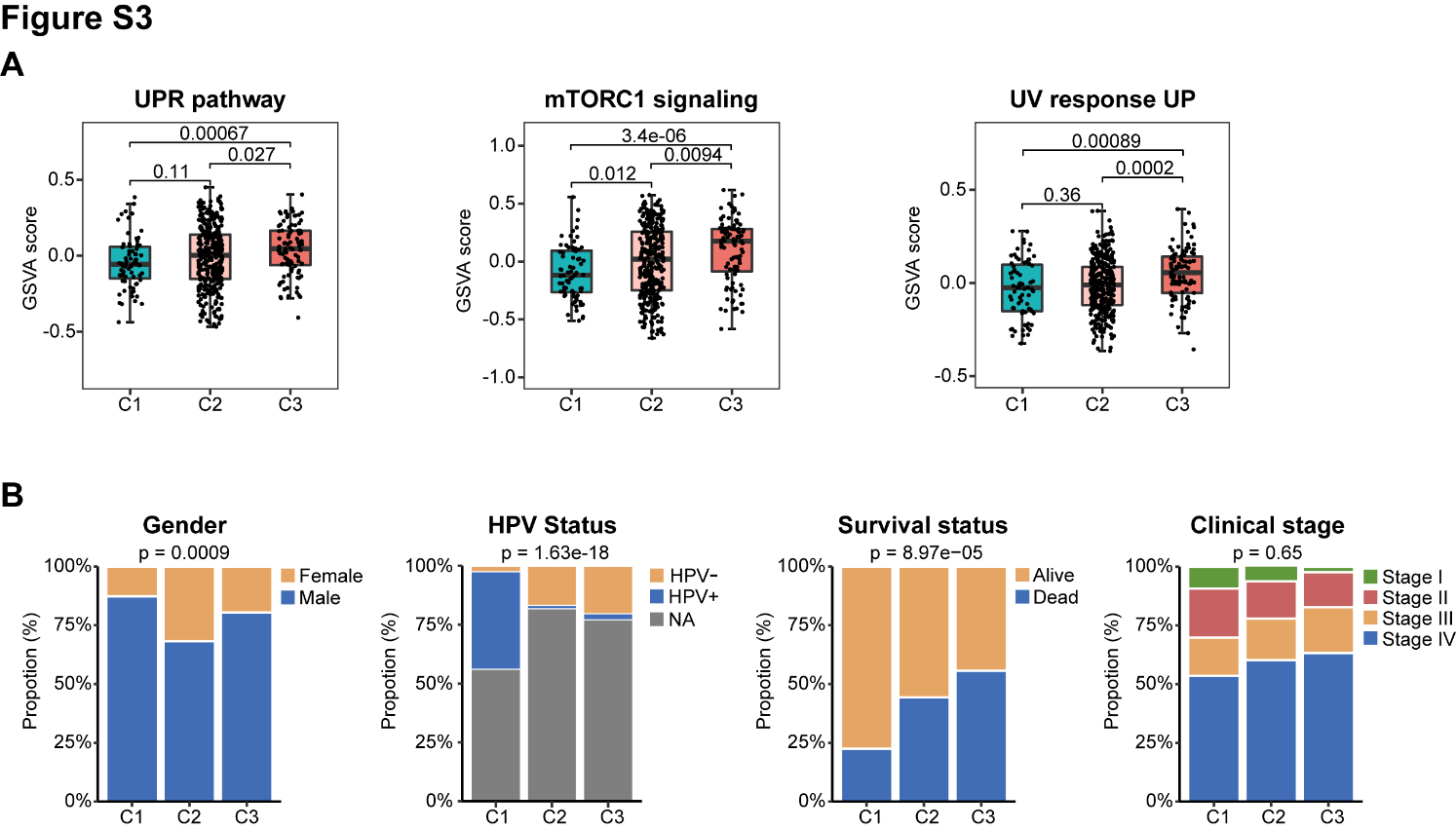
**

**Figure S3. Molecular characteristics of different HNSCC subtypes in the TCGA cohort.**

A. The distribution of pathway activity for patients in different subtypes. B. The proportion of patients with different gender, HPV status, survival status, and stages of the three subtypes.


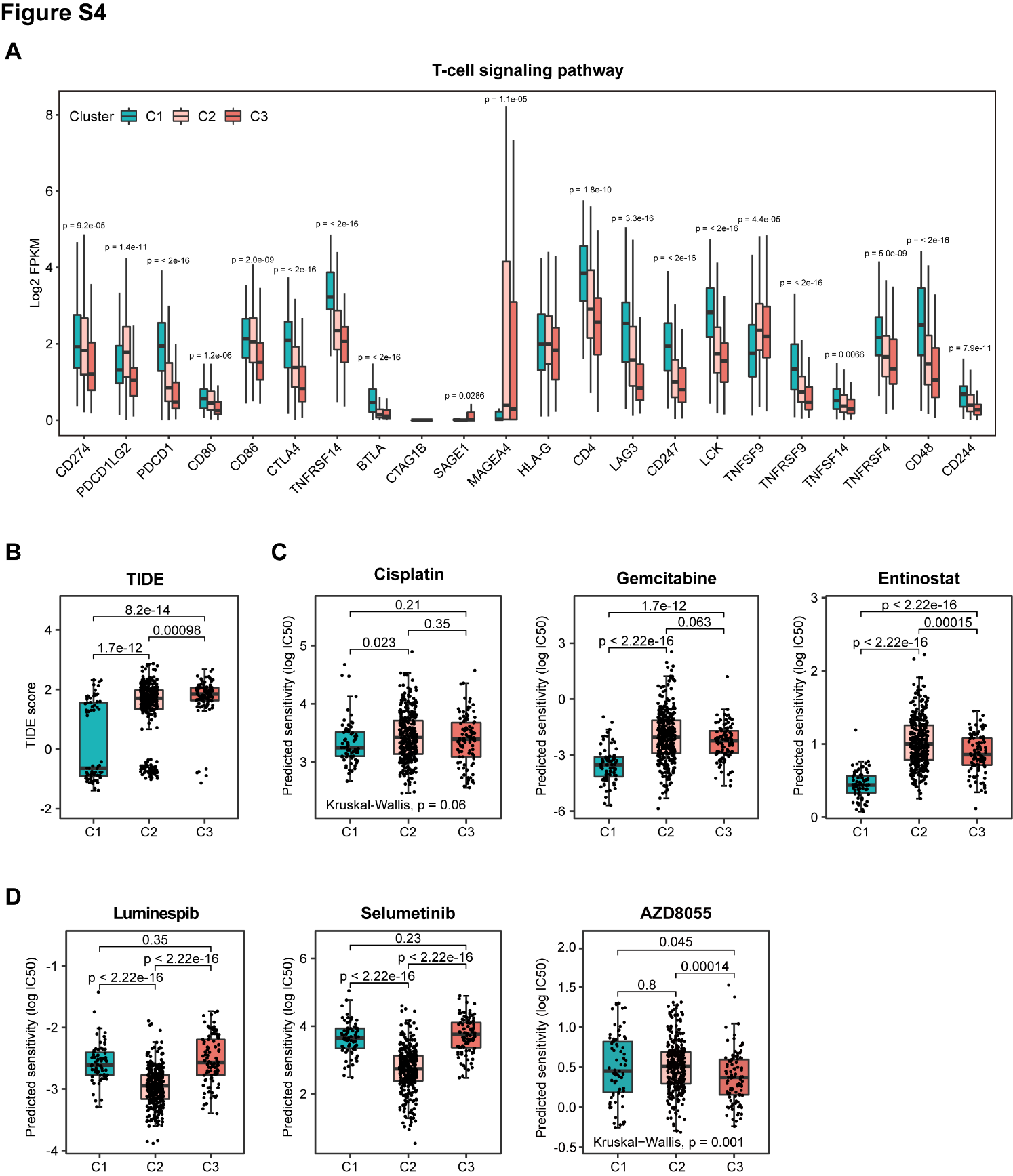


**Figure S4. Prediction of response to immunotherapeutic and therapeutic agents for different subtypes.**

A. The expression of immune checkpoint pathway for patients with HNSCC of different subtypes in TCGA cohort. B. The distribution of TIDE score in different subtypes. C and D. The box plots depict the estimated IC50 for cisplatin, gemcitabine, entinostat (C), as well as luminespib,selumetinib and AZD8055 (D). Wilcoxon rank-sum test was performed between two groups. Kruskal-Wallis test was used to conduct difference comparisons of three groups.
